# The Association of Renin-Angiotensin-Aldosterone System Inhibitors With Outcomes Among a Predominantly Ethnic Minority Patient Population Hospitalized With COVID-19: The Bronx Experience

**DOI:** 10.7759/cureus.10217

**Published:** 2020-09-03

**Authors:** Muhammad Adrish, Sridhar Chilimuri, Haozhe Sun, Nikhitha Mantri, Alla Yugay, Maleeha Zahid

**Affiliations:** 1 Pulmonary and Critical Care Medicine, Bronx Care Health System, Bronx, USA; 2 Internal Medicine, Bronx Care Health System, Bronx, USA

**Keywords:** angiotensin ii receptor blockers, coronavirus disease 2019, angiotension converting enzymes inhibitors

## Abstract

Background and objective

Angiotensin-converting enzyme inhibitors (ACE) and angiotensin II receptor blockers (ARB) are commonly used for the treatment of patients with heart disease, hypertension (HTN), and diabetes mellitus (DM). In the aftermath of the emergence of the coronavirus disease 2019 (COVID-19) pandemic, initial data raised concerns that ACE/ARB use can increase the expression of ACE2 receptors, leading to the worsening of COVID-19. However, recent studies have suggested that their use might be safe in a select subgroup of patients. We conducted a single-center retrospective study to evaluate the association of in-patient use of ACE/ARB with outcomes among a predominantly ethnic minority patient population of the inner New York City (NYC).

Methods

This was a retrospective analysis of all hospital admissions with COVID-19 from March 1, 2020, to March 31, 2020.

Results

Of the 469 patients included in the study, 91 patients (19.4%) used ACE/ARB therapy during their hospital stay and were labeled as ACE/ARB group. Patients in the ACE/ARB therapy group were older and had a higher incidence of HTN, coronary artery disease (CAD), congestive heart failure, DM, asthma, and chronic obstructive pulmonary disease. Admission D-dimer, lactate dehydrogenase (LDH), and C-reactive protein (CRP) levels were similar between the two groups, but absolute lymphocyte count (ALC) was lower in the non-ACE/ARB group (0.971 k/ul vs. 1.135 k/ul, p=0.0144). The incidence of hyperkalemia and the rise in creatinine were similar between the two groups. Univariate analysis by treatment group using the log-rank test produced significant results (p=0.0062), indicating a higher survival rate for the ACE/ARB group.

Conclusion

The use of ACE/ARB appears to be safe in all patients in whom their use is medically indicated.

## Introduction

Angiotensin-converting enzyme inhibitors (ACE) and angiotensin II receptor blockers (ARB) have been among the first-line medications in the treatment of patients with hypertension (HTN), diabetes mellitus (DM), and heart disease [1]. ACE2 receptors also play an important role in the regulation of the renin-angiotensin-aldosterone system. In the aftermath of the emergence of the coronavirus disease 2019 (COVID-19) pandemic, initial data raised concerns that ACE/ARB use may increase the expression of human ACE2 receptors, which is a cellular receptor used as an entry point for the coronavirus [2]. These findings have raised concerns about potential harm from ACE/ARB use. A study by Mancia et al. from the Lombardy region of Italy showed that ACE and ARB use was not associated with an increased likelihood of COVID-19 [3]. Similarly, a study from Wuhan in hypertensive patients with COVID-19 showed a lower risk of all-cause mortality with their use [4]. However, in this study, age- and sex-matched COVID-19-positive controls without known HTN history were used, which raises concerns for bias in the study outcomes.

New York City (NYC) has been the epicenter of COVID-19 in the US [5]. A recent study showed that of the five NYC boroughs, the Bronx has the highest proportion of ethnic minorities, poverty, and the highest rates of deaths related to COVID-19 [6]. Authors postulated that comorbid illnesses, occupational exposures, race-based structural inequalities, and socioeconomic determinants may explain this disparity. Our hospital is located in the South Bronx, which is the poorest congressional district in the nation [7], with 38% of the population living below the poverty level.

In this study, we aimed to examine the characteristics of the COVID-19-positive patients who were hospitalized during the peak of the pandemic and whether the use of ACE/ARB affected their treatment outcomes.

## Materials and methods

Study design

This was a retrospective analysis of all hospital admissions with COVID-19 from March 1, 2020, to March 31, 2020. Approval for the study was obtained from the Institutional Review Board (IRB) at the Bronx Care Health System (IRB approval number: 05 14 20 07). Written informed consent was waived by the IRB owing to the observational nature of the study as well as the setting of a rapidly evolving pandemic.

Participants and eligibility criteria

We included adult patients (aged 18 years and above) who were hospitalized with COVID-19 and had a final disposition status at the time of the study. A total of 478 COVID-19-positive patients were admitted during the study period, of which 469 patients (98.1%) had the disposition and were included in our study.

A diagnosis of COVID-19 was defined as a patient with a positive result on the nasopharyngeal swab for severe acute respiratory syndrome coronavirus 2 (SARS-CoV-2) via reverse transcription-polymerase chain reaction (RT-PCR). Nasopharyngeal swab samples were collected during the time of admission and transported in collection tubes using a preservation solution as per our laboratory protocol. Testing was performed by the RT-PCR assay as per the manufacturer’s protocol.

Diagnostic workup and management of these patients were performed based on patient’s individual needs, resource availability as well as the treating clinician’s judgment and under the guidance of existing inpatient guidelines developed by our institution based on recommendations from the Centers for Diseases Control and Prevention (CDC), and the Department of Health in NY state and NYC.

Data collection

We reviewed individual electronic medical records (EMR) of all the 469 patients included in our study. Demographic and anthropometric data such as age, gender, ethnicity, and body mass index (BMI) were collected. History of tobacco use and comorbid conditions such as HTN, DM, obstructive airway disease (OAD), cardiovascular disease (CVD), coronary artery disease (CAD), chronic kidney disease (CKD), end-stage renal disease (ESRD), and human immunodeficiency syndrome (HIV)/acquired immune deficiency syndrome (AIDS) was also collected.

Laboratory and radiological findings and trends, which were pertinent to our study, were extracted from our EMR. Only initial labs drawn on admission were collected for analysis in our study. Medication history including COVID-19-related therapy and ACE/ARB use were collected along with details of individual hospital courses such as the use of supplemental oxygen and mechanical ventilation. Patients were divided into two groups for final analysis based on the use of ACE/ARB during hospitalization.

The primary outcome measure of the study included in-hospital mortality and the need for mechanical ventilation. The length of hospital stay was selected as the secondary outcome.

Quality assessment

All the authors, at the end of data collection, verified the collected data. 

Statistical analysis

Statistical analysis was performed using STATA software version 14.2. Study participants were divided into two groups: the treatment group defined as patients who had received ACE/ARB during the course of admission versus the control group who did not receive ACE/ARB. Continuous variables were expressed as mean with standard deviations. Categorical variables were represented as counts and percentages. The Fisher’s exact test was used to compare nominal variables between two groups. Continuous variables were compared using an independent, two-tailed t-test.

For the survival time from the date of admission in the hospital to the death/discharge, univariate analysis for the two treatment groups was first conducted using the Kaplan-Meier estimates and the log-rank test. Median survival times were reported for all groups and by treatment arm together with 95% CI. A multivariate approach, a Cox model, was built with a stepwise forward procedure with a p-value of <0.1 for inclusion. The proportional hazards assumption for the final model was evaluated using the Schoenfeld residuals. For the final Cox model, the hazard ratio (HR) estimates together with 95% CI for the final model were reported. To evaluate the need for mechanical ventilation between the two groups, a logistic model was used. As the univariate approach, a logistic model was estimated with only the treatment arm as the factor. Next, the multivariate model was built using the stepwise forward method with a p-value of <0.1 for inclusion. For the final model, odds ratio (OR) estimates together with 95% CI were reported. In order to assess the predictive ability of the final logistic model, the receiver operating characteristic (ROC) curve was plotted and the area under the curve calculated.

## Results

Of the 469 patients included in the study, 91 patients (19.4%) used ACE/ARB therapy during their hospital stay and were labeled as ACE/ARB group. Of note, 96.7% of the ACE/ARB group patients and 88.8% of the non-ACE/ARB group belonged to Hispanic/Latino and African American communities. Patients in ACE/ARB therapy group were older (64.6 years vs. 58.26 years, p=0.0008) and had higher incidence of HTN (86.8% vs. 51.3%, p=0.0001), CAD (19.7% vs. 10.6%, p=0.02), congestive heart failure (19.7% vs. 10%, p=0.0178), DM (60.4% vs. 40.2%, p=0.0046), asthma (25.3% vs. 15.9%, p=0.0458), and chronic obstructive pulmonary disease (19.7% vs. 10.6%, p=0.02) compared to non-ACE/ARB group (Table 1).

**Table 1:** Admission demographics, laboratory findings, and outcomes ACE: angiotensin-converting enzyme inhibitors; ARB: angiotensin II receptor blockers; ALC: absolute lymphocyte count; ANC: absolute neutrophil count; COPD: chronic obstructive pulmonary disease; ESRD: end-stage renal disease; SD: standard deviation

Variables	ACE/ARB group (n=91)	Non-ACE/ARB group (n=378)	P-value
Age in years, mean (SD)	64.6 (12.13)	58.26 (16.8)	0.0008
Sex, n (%)			0.4769
Female	40 (44%)	150 (39.7%)	
Male	51 (56%)	228 (60.3%)	
BMI ≥30, n (%)	41 (45.1%)	163 (43.1%)	0.8139
Race, n (%)			
African American	25 (27.5%)	106 (28%)	1.000
Hispanics/Latino	63 (69.2%)	230 (60.8%)	0.1492
White/Caucasian	1 (1.1%)	6 (1.6%)	1.000
Others	2 (2.2%)	36 (9.5%)	0.0181
Comorbidities, n (%)			
Current or ex-smoker	25 (27.5%)	104 (27.5%)	1.000
Hypertension	79 (86.8%)	194 (51.3%)	0.0001
Coronary artery disease	18 (19.7%)	40 (10.6%)	0.02
Congestive heart failure	18 (19.7%)	38 (10%)	0.018
Diabetes mellitus	55 (60.4%)	152 (40.2%)	0.0046
Asthma	23 (25.3%)	60 (15.9%)	0.0458
COPD	18 (19.7%)	40 (10.6%)	0.0213
Chronic kidney disease	11 (12.1%)	41 (10.8%)	0.7121
ESRD	5 (5.5%)	32 (8.5%)	0.5147
HIV infection/AIDS	5 (5.5%)	32 (8.5%)	0.5147
Mean duration of symptoms before admission in days (SD)	6.10 (4.75)	5.45 (4.28)	0.2273
Initial laboratory on admission, median (SD)			
Absolute neutrophil count, k/ul	5.253 (3.040)	5.988 (3.340)	0.0575
Absolute lymphocyte count, k/ul	1.135 (0.773)	0.971 (0.505)	0.0144
ANC/ALC ratio	6.342 (5.391)	8.032 (7.320)	0.0401
D-dimer, ng/ml	1775.5 (5858.5)	1921 (6682.88)	0.8776
Lactate dehydrogenase, unit/L	513.27 (416.12)	559 (367.79)	0.3662
C-reactive protein, mg/L	143.72 (130.34)	109.42 (110.1)	0.0562
Admission chest X-ray, n (%)			
Normal	11 (12.1%)	50 (13.2%)	0.8634
Alveolar/interstitial infiltrates	77 (84.6%)	316 (83.6%)	0.8754
Pleural effusion	0 (0%)	1 (0.2%)	1.000
No imaging	3 (3.3%)	11 (2.9%)	0.7404
Adverse events, n (%)			
Sodium serum ≥145 mEq/L	10 (11%)	45 (11.9%)	1.000
Sodium serum ≥155 mEq/L	3 (3.3%)	9 (2.4%)	0.7095
Potassium serum ≥5.5 mEq/L	18 (19.8%)	71 (18.8%)	0.8816
Creatinine serum ≥1.5 mg/dl	34 (37.4%)	140 (37%)	1.000
Oxygen therapy, n (%)			
Low-flow oxygen	41 (45%)	165 (43.6%)	0.815
High-flow oxygen	1 (1.1%)	17 (4.5%)	0.2195
Invasive mechanical ventilation	36 (39.5%)	150 (39.7%)	1.00
Medications, n (%)			
Hydroxychloroquine	74 (81.3%)	299 (79.1%)	0.7723
Anti-retrovirals	7 (7.7%)	29 (7.7%)	1.000
Systemic steroids	18 (19.8%)	82 (21.7%)	0.7762
Length of hospital stay in days, mean (SD)	9.85 (5.25)	7.46 (4.23)	0.0001
Mortality, n (%)	32 (35.2%)	138 (36.5%)	0.9034

There was no difference between the two groups in the mean duration of symptoms before admission to the hospital; 84.6% of patients in the ACE/ARB group and 83.6% of patients in the non-ACE/ARB group had radiographic evidence of pneumonia at the time of admission. Admission D-dimer, lactate dehydrogenase (LDH), and C-reactive protein (CRP) levels were similar between the two groups. Non-ACE/ARB group had lower absolute lymphocyte count (0.971 k/ul vs. 1.135 k/ul, p=0.0144) and higher absolute neutrophil count to absolute lymphocyte count (ANC/ALC) ratio compared to the ACE/ARB group.

**Figure 1:**
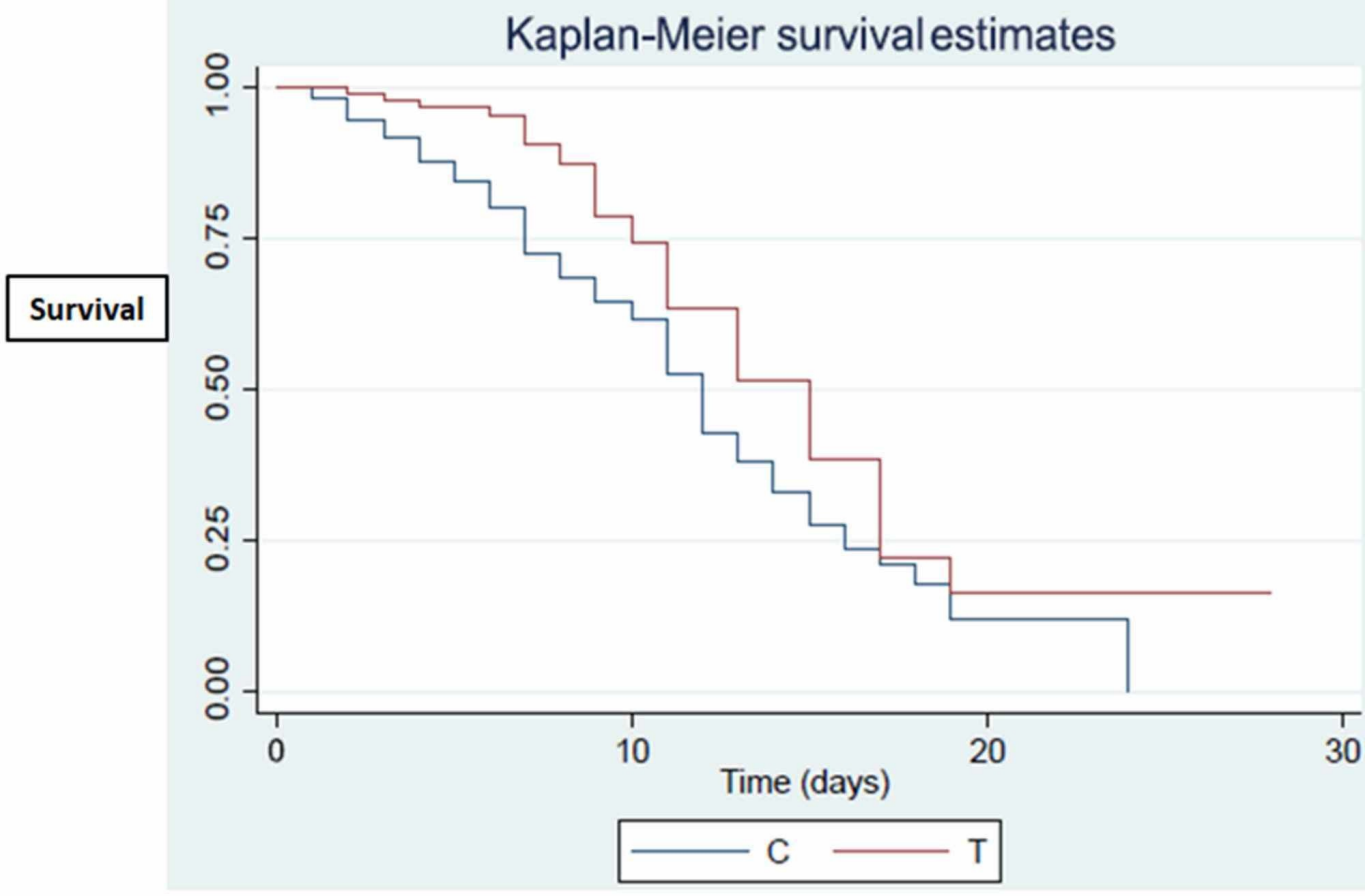
Univariate analysis by treatment group using log-rank test The chart indicates a higher survival rate for ACE/ARB group (denoted T) compared to the non-ACE/ARB group (denoted C) ACE: angiotensin-converting enzyme inhibitors; ARB: angiotensin II receptor blockers

There was no difference in the incidence of hyperkalemia or hypernatremia between the two groups. There was a statistically significant difference in the length of hospital stay between the ACE/ARB group and the control group (median 9.31 days vs. 6.9 days respectively, p=0.0001). No difference was observed in terms of the need for mechanical ventilation or mortality between the two groups. Univariate analysis by treatment group using a log-rank test produced significant results (p=0.0062) indicating the higher survival time from admission to disposition (death/discharge) for the ACE/ARB group (Figure 1).

The median survival time for the ACE/ARB group was 15 days (95% CI: 11-17), whereas it was 12 days (95% CI: 11-13) for the non-ACE/ARB group. In the multivariate approach Cox model for the survival time from date of admission in the hospital, significant effects were observed for age, history of CAD, history of tobacco product use, and admission labs including ANC/ALC ratio, LDH, and CRP. Of these parameters, only the presence of CAD was associated with higher survival (Table 2).

**Table 2:** Cox model for survival from the date of admission in the hospital ALC: absolute lymphocyte count; ANC: absolute neutrophil count; CAD: coronary artery disease; CI: confidence interval; CRP: C-reactive protein; HR: hazard ratio; LDH: lactate dehydrogenase; SE: standard error

Variables	HR	SE	Z-score	P-value	95% CI
Age	1.0429	0.0083	5.2800	0.0000	1.0268-1.0592
CAD	0.5714	0.1535	-2.0800	0.0370	0.3375-0.9673
Smoking	1.7716	0.3393	2.9900	0.0030	1.2171-2.5787
ANC/ALC ratio	1.0258	0.0095	2.7700	0.0060	1.0075-1.0445
Admission LDH	1.0007	0.0002	2.9800	0.0030	1.0002-1.0011
Admission CRP	1.0018	0.0006	3.1100	0.0020	1.0007-1.0029

**Figure 2:**
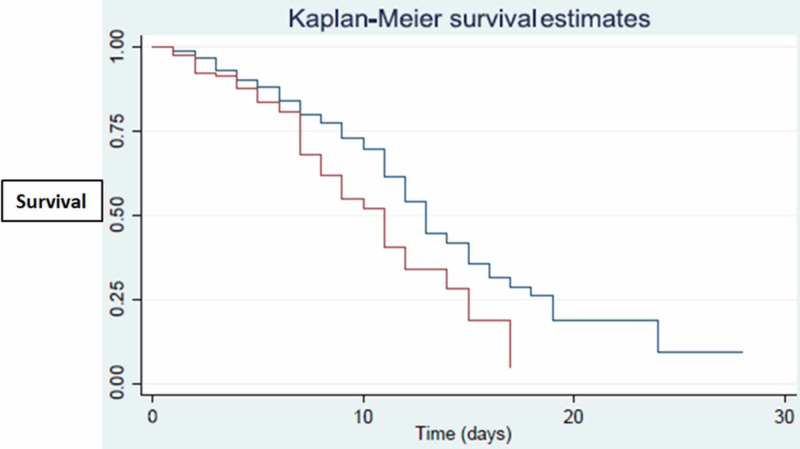
Kaplan-Meier estimates for the survival curve for the smoking history variable [Red line (T) denotes smokers, blue lines (C) denotes non-smokers]

An HR estimate of 1.04 for the age correlated with a 4% increase in the risk of death with every single year of age for the patient. Mortality also increased by 3% for every unit increase in the ANC/ALC ratio. Patients with a history of tobacco product use had an HR of 1.77. The history of the tobacco use variable was further represented using the Kaplan-Meier plot, which showed a significant association with mortality (p=0.0013) (Figure 2).

For the logistic model for the need for mechanical ventilation, a significant effect was observed for the following variables: age, history of DM, history of HIV/AIDS, and admission labs including serum LDH and CRP (Table 3).

**Table 3:** Logistic model for the need for mechanical ventilation ALC: absolute lymphocyte count; ANC: absolute neutrophil count; CI: confidence interval; CRP: C-reactive protein; DM: diabetes mellitus; HIV/AIDS: human immunodeficiency virus/acquired immunodeficiency syndrome; HR: hazard ratio; LDH: lactate dehydrogenase; SE: standard error

Variables	HR	SE	Z-score	P-value	95% CI
Age	1.039786	.0101599	3.99	0.000	1.020062
DM	1.915057	.5196139	2.39	0.017	1.125186
HIV/AIDS	3.529581	2.073619	2.15	0.032	1.11595
ANC/ALC ratio	1.040059	.0207594	1.97	0.049	1.000157
Admission LDH	1.001819	.0005356	3.40	0.001	1.00077
Admission CRP	1.002296	.0011818	1.94	0.052	.9999823
Intercept	.0105184	.0074026	-6.47	0.000	.0026478

**Figure 3:**
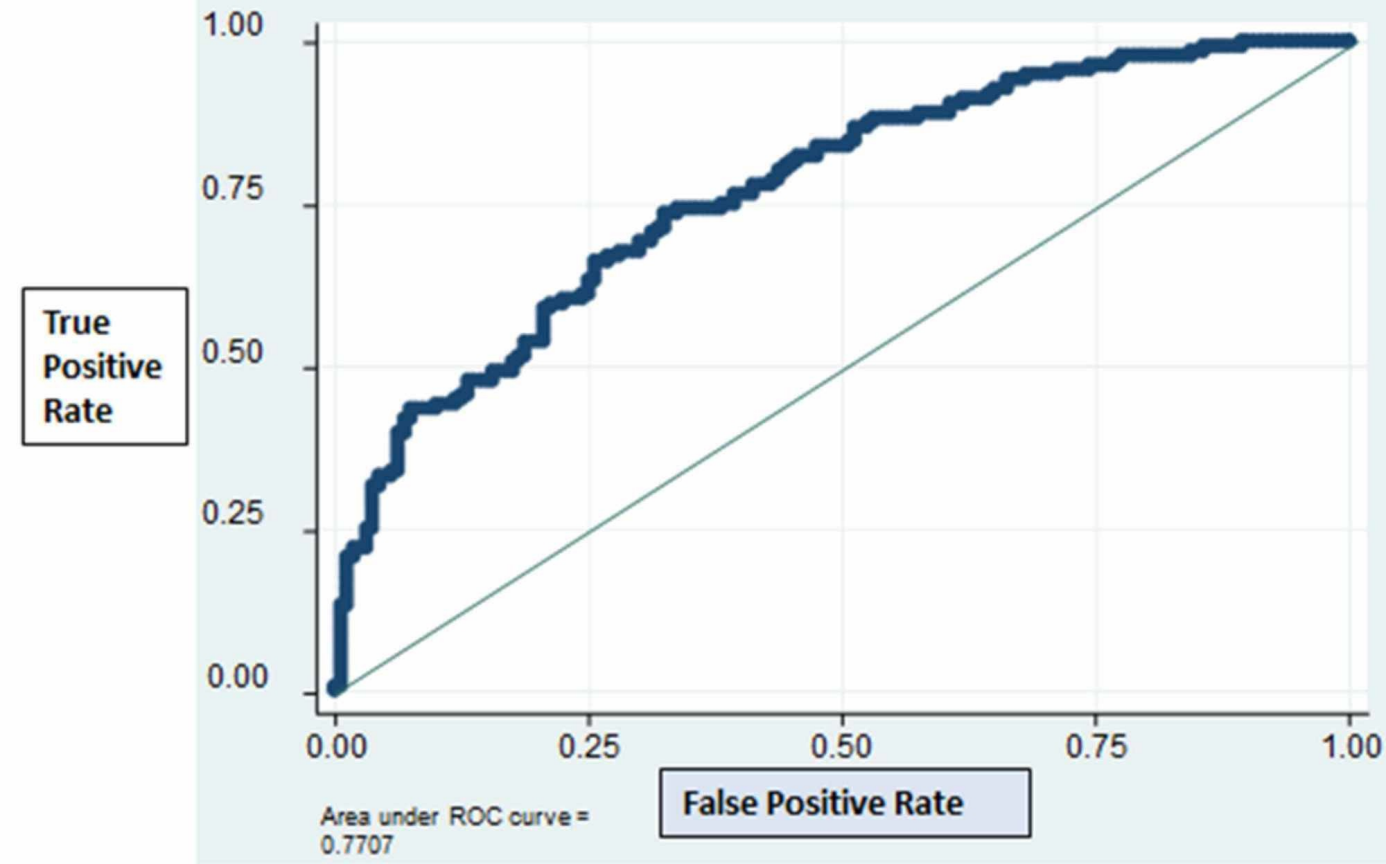
Receiver operator curve for the final logistic model

There was no significant effect for the ACE/ARB group in the adjusted model; hence the group variable was not included (p=0.682). A ROC curve for the final logistic model is presented in Figure 3.

The area under the curve was 0.77, which indicated an acceptable predictive power.

## Discussion

This is the first study to look at the role of ACE/ARB use in a predominantly ethnic minority population in the US. The main findings of our study can be summarized as follows: there was no difference in terms of the need for mechanical ventilation or mortality between the groups in a univariate analysis. Using the log-rank test, univariate analysis was performed for the treatment group, which produced significant results indicating a higher survival rate for the ACE/ARB group (Figure 1). The median survival time for the ACE/ARB group was longer than the non-ACE/ARB group. In the multivariate approach Cox model for the survival time from the date of admission, age, history of CAD, history of tobacco product use, ANC/ALC ratio, and serum LDH and CRP were associated with survival. In the logistic model for the need for mechanical ventilation, a significant effect was observed for age, history of DM, history of HIV/AIDS, and serum LDH and CRP. Our study also highlights the important variations in baseline demographics that might explain differences in outcomes for ethnic minorities hospitalized with COVID-19. In addition to poverty, our patients had some of the highest incidences of obesity (41%), HTN (58%), DM (55%), and CAD (18%) compared to other studies [4, 8-10]. Our study patients were also sicker as close to 40% of them required mechanical ventilation during the hospital stay.

Despite the great knowledge gained over the past few months regarding the clinical characterization and diagnosis of COVID-19, it remains a dynamic disease process of which our understanding is still evolving. This is a heterogeneous disease that continues to affect patients of all age groups and not just older patients. SARS-CoV-2 entry into the cells is guided by the expression of ACE2 receptors on the cellular membranes [11], and such a combination can abolish ACE2 enzyme effects, an enzyme that converts angiotensin II (pro-inflammatory/pro-coagulant) to angiotensin 1-7 (anti-inflammatory/anti-coagulant) [12]. After binding to ACE2 receptors, SARS-CoV-2 induces down-regulation of ACE2 in host cells, which leads to increased angiotensin II concentration, which is an effector peptide of the renin-angiotensin system [13,14]. In animal studies, angiotensin II has been shown to increase levels of various inflammatory markers including tumor necrosis factor α, gamma interferon, interleukin (IL) 6, IL-1β, IL-17, and IL-23 [15]. Animal studies have also shown that lung ACE2 levels in the older rats were lower compared to younger rats [16]. As such, COVID-19 infection-induced further down-regulation of ACE2 receptors in older patients may lead to an overall severe inflammatory state. Treatment with ACE and ARB down-regulates the inflammatory cytokines by decreasing the production of angiotensin II, thereby increasing the expression on the ACE2 receptor [17].

Our study has several limitations. Firstly, this was a retrospective, single-center study, and the interpretation of our study results can be limited due to sample size or selection bias. Second, we did not include the data on ACE/ARB use prior to admission, as this information was not available for many of our study patients. Thirdly, we did not measure levels of inflammatory markers. Our study did account for the severity of illness as expressed by the escalating need for oxygen and mechanical ventilation. And, finally, we did not perform a propensity score-based matching analysis to minimize the effects of confounders. Instead, we performed multivariate approach Cox models with stepwise forward analysis to address these confounders.

We believe further prospective studies are needed to examine whether the ACE/ARB use is beneficial for all COVID-19 patients.

## Conclusions

In this retrospective single-center study involving a predominantly ethnic minority population, the use of ACE/ARB for COVID-19 illness was found to be safe with no added risk for renal failure or electrolyte abnormalities. The ACE/ARB therapy did not lead to a higher need for mechanical ventilation and mortality. Based on our study findings, we recommend the continued use of ACE/ARB in acute COVID-19 infections for patients in whom their use is otherwise medically indicated. Further prospective studies are needed to validate our study findings.
